# Low Back Pain and Disability In Activities of Daily Living Mmong Middle-aged And Older Adults In China: A Cohort Study

**DOI:** 10.1093/geroni/igaf122.3217

**Published:** 2025-12-31

**Authors:** Bo Liang, Xiaoxuan Liang, Gong Chen

**Affiliations:** Peking University, Beijing, Beijing, China (People’s Republic); Peking University, Beijing, Beijing, China (People’s Republic); Peking University, Beijing, Beijing, China (People’s Republic)

## Abstract

Chronic diseases are associated with disability in activities of daily living (ADL), but the relationship between low back pain (LBP) and the risk of ADLs disability remains unclear. This study aimed to estimate the effect of LBP on ADLs disability and explore variations in the risk across gender and age among middle-aged and older adults in China. Participants from the China Health and Retirement Longitudinal Study (CHARLS) 2015 were followed up to assess disabilities of basic ADL (BADL) and instrumental ADL (IADL) in 2018 and 2020. The cohorts comprised 19,223 participants aged ≥45 with intact BADL, and 18,226 with intact IADL, respectively. Cox proportional hazards model was employed to investigate the association between LBP and BADL and IADL disabilities. The incidence rates for BADL and IADL disabilities were 17.81 and 38.32 per 1,000 person-years, respectively. Compared to participants without LBP, those with either back or waist pain had a 64% (HR = 1.64, 95%CI: 1.43-1.87) increased risk of BADL disability, and those with both types of pain showed a 105% (HR = 2.05, 95%CI: 1.79-2.35) increased risk. Similarly, those with only back or waist pain, and pains combined presented a 61% (HR = 1.61, 95%CI: 1.46-1.78) and 122% (HR = 2.22, 95%CI: 2.01-2.45) increased risk of IADL disability, respectively. Subgroup analyses indicated that LBP increased the risk of ADLs disabilities across genders and age groups. Therefore, the increased risk of disabilities in BADL and IADL may be affected by LBP among middle-aged and older Chinese. Early prevention of LBP occurrence can delay or reduce ADLs disabilities risk.

